# Spatial and temporal analysis of HIV clinical outcomes in Florida reveals counties with persistent racial and ethnic disparities during 2012-2019

**DOI:** 10.1186/s12889-024-17944-w

**Published:** 2024-03-09

**Authors:** Shannan N Rich, Yiyang Liu, Rebecca Fisk-Hoffman, Yi Zheng, Hui Hu, Emma E Spencer, Robert L Cook, Mattia Prosperi

**Affiliations:** 1 Department of Epidemiology, Colleges of Public Health and Health Professions and Medicine, University of Florida, Gainesville, FL 32603 USA; 2 Channing Division of Network Medicine, Department of Medicine, Brigham and Women’s Hospital and Harvard Medical School, Boston, MA 02115 USA; 3 Florida Department of Health, Division of Disease Control and Health Protection, Bureau of Communicable Diseases, HIV/AIDS Section, Tallahassee, FL 32399 USA

**Keywords:** HIV, Spatial-temporal epidemiology, Clinical outcomes, Racial disparities, HIV in the South

## Abstract

**Background:**

Racial/ethnic disparities in the HIV care continuum have been well documented in the US, with especially striking inequalities in viral suppression rates between White and Black persons with HIV (PWH). The South is considered an epicenter of the HIV epidemic in the US, with the largest population of PWH living in Florida. It is unclear whether any disparities in viral suppression or immune reconstitution—a clinical outcome highly correlated with overall prognosis—have changed over time or are homogenous geographically. In this analysis, we 1) investigate longitudinal trends in viral suppression and immune reconstitution among PWH in Florida, 2) examine the impact of socio-ecological factors on the association between race/ethnicity and clinical outcomes, 3) explore spatial and temporal variations in disparities in clinical outcomes.

**Methods:**

Data were obtained from the Florida Department of Health for 42,369 PWH enrolled in the Ryan White program during 2008-2020. We linked the data to county-level socio-ecological variables available from County Health Rankings. GEE models were fit to assess the effect of race/ethnicity on immune reconstitution and viral suppression longitudinally. Poisson Bayesian hierarchical models were fit to analyze geographic variations in racial/ethnic disparities while adjusting for socio-ecological factors.

**Results:**

Proportions of PWH who experienced viral suppression and immune reconstitution rose by 60% and 45%, respectively, from 2008-2020. Odds of immune reconstitution and viral suppression were significantly higher among White [odds ratio =2.34, 95% credible interval=2.14-2.56; 1.95 (1.85-2.05)], and Hispanic [1.70 (1.54-1.87); 2.18(2.07-2.31)] PWH, compared with Black PWH. These findings remained unchanged after accounting for socio-ecological factors. Rural and urban counties in north-central Florida saw the largest racial/ethnic disparities.

**Conclusions:**

There is persistent, spatially heterogeneous, racial/ethnic disparity in HIV clinical outcomes in Florida. This disparity could not be explained by socio-ecological factors, suggesting that further research on modifiable factors that can improve HIV outcomes among Black and Hispanic PWH in Florida is needed.

**Supplementary Information:**

The online version contains supplementary material available at 10.1186/s12889-024-17944-w.

## Background

The Southern region within the United States (US) is considered an epicenter of the HIV epidemic, with the largest population of people with HIV (PWH) living in Florida. There are over 117,000 Floridians with HIV, with a disproportionate number of these individuals (45%) identifying as Black (27% White, 25% Hispanic/Latinx) [1]. The Ending the HIV Epidemic in the US (EHE) initiative aims to reduce HIV incidence by 90% by 2030 [2]. The EHE lists 57 priority jurisdictions across the US, seven of them are in Florida. A key pillar of the EHE is getting PWH to achieve and sustain viral suppression quickly. This has been made possible by the advent of combination antiretroviral therapy (ART) enables PWH to medically manage their infection and effectively eliminate the risk of transmission [3, 4]. However, in Florida it is estimated that less than two-thirds of PWH have achieved viral suppression [5]. Research is needed to identify actionable factors to improve HIV clinical outcomes in this high incidence and prevalence setting.

Racial/ethnic disparities throughout the HIV care continuum have been well documented in the US [6–9], with especially striking inequalities in viral suppression rates between White and Black PWH [10]. For example, based on the National HIV Surveillance System, an estimated 36.1% of Black PWH aged 13-29 achieved sustained viral suppression, lower than the 46.7% observed in Hispanic/Latinx PWH and 50.8% in White PWH within the same age group [11]. Data from Florida indicate that it takes longer for Black PWH to achieve viral suppression than White and Hispanic PWH during the first several years after HIV diagnosis [12] . These disparities are partially explained by individual-level factors, but the remaining disparities could be attributed to socio-ecological factors. Factors including local healthcare and transportation infrastructure, the health behaviors of those around them, and the local economic context can influence health behaviors, including those that influence HIV health outcomes [13–16]. In Florida, one cross-sectional analysis of the state’s enhanced HIV/AIDS reporting system (eHARS) in 2015 revealed that Black and Latinx PWH more likely than White PWH to have unsuppressed HIV viral load (47% among Black PWH, 37% among Latinx PWH, and 32% among White PWH) [13]. After individual and community factors were controlled, the disparity between Latinx and White PWH reduced precipitously to 7%, however, Black PWH was still 55% more likely not to have viral suppression [13]. Immune reconstitution on ART, defined as a restoration of CD4 T cell counts soon after initiation of ART [17], is another clinical outcome important for overall prognosis, yet few studies have investigated the presence of racial/ethnic disparities for this outcome. One study conducted among PWH in Texas reported that the rate of immune recovery was not significantly different by race/ethnicity [18] though more studies are needed to confirm this finding.

Additionally, geographic variation in HIV burden by race has been documented [19]. For example, an analysis using HIV mortality data from Florida and found that disparities between White and Black PWH widened over time before stabilizing [20]. However, that study did not examine spatial variation in HIV mortality by race. Spatial-temporal variations in racial disparities have been found in Florida for non-HIV disease burdens [21–23]. For instance, greater racial disparities in hypertensive disorders of pregnancy burden have been found to occur in the North Central Florida counties throughout the past decade [23]. However, little is known about the spatial-temporal variation in racial/ethnic disparities in HIV clinical outcomes in Florida. Since much of the HIV response is conducted at the local level, a better understanding of HIV clinical outcomes by race across space and time could guide targeted intervention to improve HIV care outcomes and reduce racial/ethnic disparities.

As Florida seeks to achieve the EHE goals, it is important to assess the progress made in reaching HIV-related health targets and identify populations that are lagging behind and where these lags are occurring within the state. The objectives of this study were to 1) investigate longitudinal trends in clinical outcomes among PWH in Florida between 2012 and 2019, 2) examine the impact of socio-ecological factors on the association between race/ethnicity and HIV clinical outcomes, 3) explore spatial and temporal variations in HIV clinical outcome by race/ethnicity.

## Methods

### Ethics statement

This secondary analysis and a waiver of informed consent were approved by the institutional review boards at the University of Florida (IRB202001385) and the Florida Department of Health (FDOH), as the data were originally collected for public health activities and purposes. The data are made available upon reasonable request by submitting an application to the FDOH Bureau of Communicable Diseases.

### Data sources

Deidentified, individual-level demographic and clinical data were obtained from the FDOH's enhanced HIV/AIDS Reporting System (eHARS) and CAREWARE for the period 2008–2020. We focused on PWH receiving HIV care through the Ryan White Program (*n*=42,369) as this cohort receives services sponsored by the FDOH and maintains a robust database of longitudinal clinical outcomes. The Florida Ryan White Program has the following eligibility criteria: must be HIV positive, live in Florida, cannot be receiving the same services from Medicaid (or other insurance), and must be low-income (at or below 400 percent of the Federal Poverty Level). ​ Florida Ryan White enrollment forms are available in English, Spanish, and Haitian Creole. Citizenship or proof of legal residence is not a requirement for receiving Ryan White services. The eHARS and CAREWARE data was linked with the publicly available County Health Rankings (CHR) data (https://www.countyhealthrankings.org/) using county of residence at the time of diagnosis in eHARS to extract socio-ecological variables (described below).

### Measurements

#### Clinical outcomes

Incomplete immune reconstitution was defined as CD4 T cell counts below 500 cells/μL and viral suppression was defined as HIV RNA viral load less than 200 copies/mL after initiation of ART. We permitted one of each test result per year when available, selecting the latest result in cases of multiple results per year, for all persons included in the study throughout the observation period.

#### Individual-level covariates

Age (years) was determined at the time of diagnosis. Years since HIV diagnosis was calculated from age at HIV diagnosis, age as of 2019, and clinical outcome measurement year. Race/ethnicity was categorized as Hispanic/Latinx of any race (referred to as Hispanic hereafter), non-Hispanic Black/African American (referred to as Black hereafter), non-Hispanic White, or non-Hispanic other/unknown. Transmission risk group was coded as men who have sex with men (MSM), heterosexual, individuals with intravenous drug use (IDU), MSM with IDU (MSM+IDU), those who acquired HIV through mother-to-child transmission (perinatal), or other/unknown. All individual-level covariates were available with the above categorizations from eHARS.

#### Socio-ecological variables

From the CHR, the following county-level socio-ecological variables assessed included: social economic factors rank, quality of life rank​, health behaviors rank, physical environment rank, county averaged years of potential life lost​, county averaged mentally unhealthy days, low birthweight percentage, percentage of adults reporting binge or heavy drinking, adult smoking percentage​, uninsured percentage, adult obesity percentage, adult diabetes percentage, 65 years and over percentage, rural percentage, ratio of population to primary care physicians, high school graduation rate, crime rate, income inequality ratio, ​air pollution particulate matter, and median household income​. These variables were available yearly for all Florida counties within the timeframe of interest. As noted above these variables were linked to individuals by county at the time of diagnosis and year. Pearson’s correlation coefficient was calculated for each pair combination of socio-ecological variables. For highly correlated pairs, only the variable with the strongest association with the outcome was retained.

#### Statistical approach

All analyses were conducted in R statistical programming software (https://www.r-project.org/), version 4.1.2, using the following packages: gee and INLA. Firstly, we calculated the proportion of people who achieved immune reconstitution and viral suppression among those who had at least one lab test result each year between 2012 and 2019, both overall and within specific race/ethnicity groups. To assess the statistical significance of the trends over time, we applied bivariate linear regression models with year as the independent variable.

To assess the relationship between race/ethnicity and HIV clinical outcomes, we constructed Integrated Nested Laplace Approximation models using individual-level factors, which included race/ethnicity, age at HIV diagnosis, years since HIV diagnosis, transmission risk group, and measurement year. Subsequently, we introduced socio-ecological variables described earlier into the model, to assess how the relationships between race/ethnicity and HIV clinical outcomes changed. We also incorporated a mixed-effects component into the model to account for repeated measures. We calculated odds ratios (ORs) and their corresponding 95% credible intervals (95% CI) and compared them before and after the inclusion of socio-ecological variables in the model.

#### Spatial and temporal analysis

To assess the temporal changes on the effect of race/ethnicity on the odds of immune reconstitution and viral suppression, we fit repeated measures GEE models, stratifying by year of assessment. To assess the spatial associations with racial/ethnic disparities, clinical outcome variables were treated as count variables and aggregated by race/ethnicity and year at the county level. Poisson Bayesian hierarchical models were fit to assess the spatial patterns of racial/ethnic disparities at the county-level, using county of residence at the time of diagnosis. As this spatial model can only handle binary variables, race/ethnicity was collapsed into “non-Hispanic Black” and all other racial/ethnic groups (labeled “non-Black”, even though this group includes a very small proportion of Hispanic Blacks). Relative risks and 95% confidence intervals were calculated. ​Details about the spatial and temporal analysis have been described previously [23].

## Results

Data were available for 42,369 PWH in Florida between 2008 and 2020. Due to data quality concerns with reporting changes and incomplete data during the period prior to 2012 and after 2019, we restricted our subsequent analyses to this time frame. Most of the population was aged 45-54 (26.5%) or 55–64 years (28.5%), male (68.0%), non-Hispanic Black (52.2%), and heterosexual (39.7%) or men who have sex with men (42.2%) (Table 1).

**Table 1:** Characteristics of persons with HIV in Florida who received laboratory assessments by year, *n*=42,369

Sample characteristics	**n (%)**
**Age at diagnosis**	
<25	894 (2.1%)
25-34	6,054 (14.3%)
35-44	7,509 (17.8%)
45-54	11,212 (26.5%)
55-64	12,074 (28.5%)
65+	4,626 (10.9%)
**Sex at birth**	
Female	13,573 (32.0%)
Male	28,796 (68.0%)
**Race**	
Non-Hispanic Black	22,102 (52.2%)
Hispanic	8,272 (19.5%)
Non-Hispanic White	11,063 (26.1%)
Other/unknown	932 (2.2%)
**Transmission risk group**	
Heterosexual	16,836 (39.7%)
IDU	3,906 (9.2%)
MSM	17,871 (42.2%)
MSM&IDU	1,710 (4.0%)
Other/Unknown	1,428 (3.4%)
Perinatal	618 (1.5%)

Results are presented as frequencies (percentages). Abbreviations: MSM, men who have sex with men; IDU, intravenous drug use

**Fig. 1:**
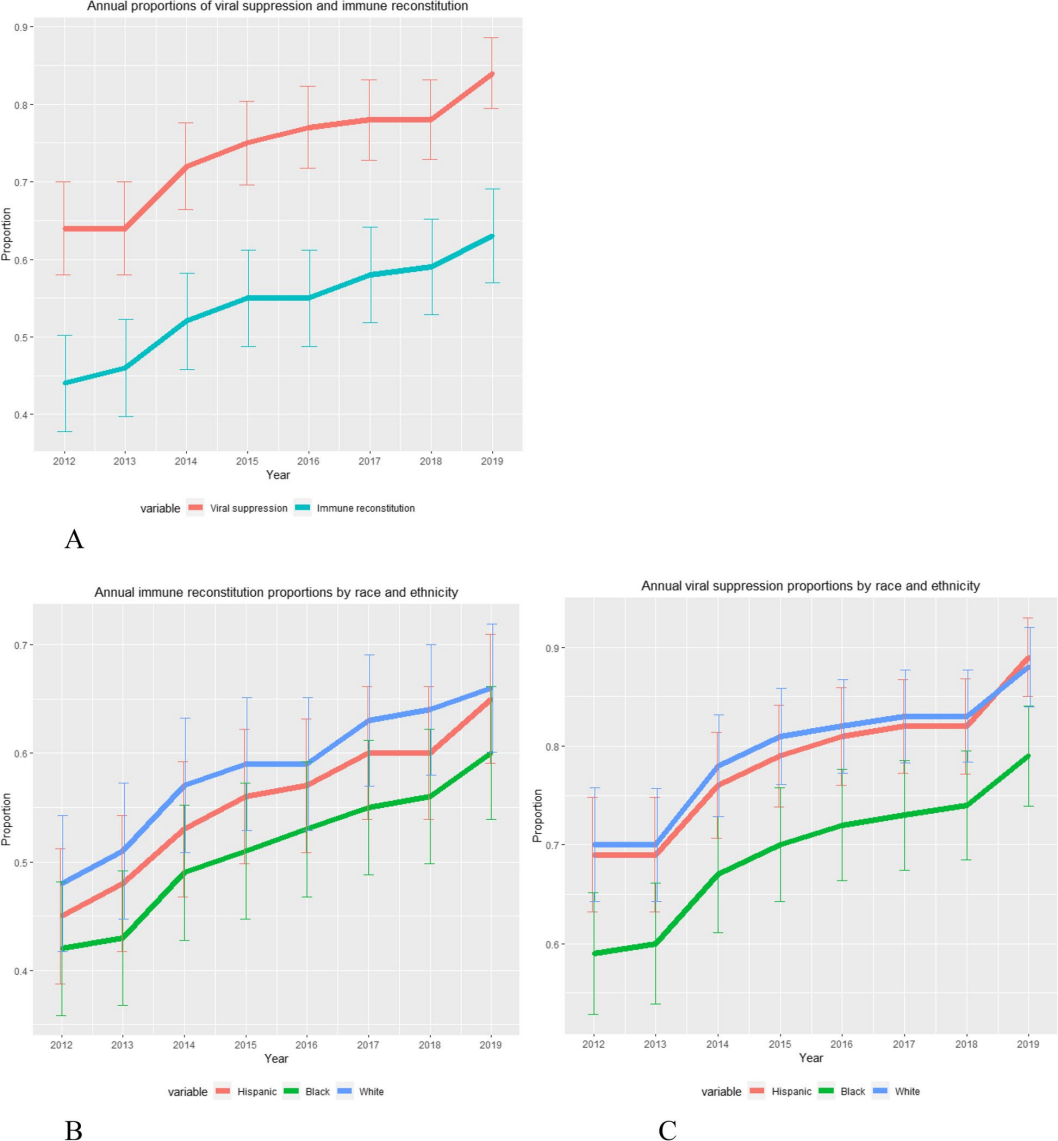
Longitudinal proportions of immune reconstitution and viral suppression overall (Panel **A**) and by race/ethnicity (Panels **B** and **C**) in Florida annually, from 2012 – 2019

The proportion of PWH with suppressed viral loads in this cohort rose from about 64% in 2012 to 84% in 2019. Similarly, the proportion of PWH who experienced immune reconstitution also rose from about 44% in 2012 to about 63% in 2019 (Fig. 1, Panel A). The increase over time was statistically significant for both outcomes (*P*<0.001). The levels of CD4 reconstitution by transmission risk group were overlapping throughout the study period in general, however, from 2015 and onward, we observed a lower proportion of individuals with immune reconstitution within the perinatal group compared to the other risk groups (data not shown). Although the levels of CD4 reconstitution improved significantly throughout the study period for all racial/ethnic and ethnic groups, the proportions were consistently higher among White PWH compared to Hispanic and Black PWH (Fig. 1, Panel B). Similarly, we observed significant improvement in viral suppression for all race/ethnicity groups over time, however, the proportions were consistently higher among White and Hispanic PWH compared to Black PWH (Fig. 1, Panel C).

### Socio-ecological findings

After controlling for individual-level covariates, the odds of both immune reconstitution and viral suppression were significantly higher among Hispanic (odds ratio [OR]= 1.70; 95% 95% credible interval [CI]=1.54-1.87 for immune reconstitution; OR= 2.18; 95% CI=2.07-2.31 for viral suppression) and White PWH (OR=2.34; 95% CI=2.14-2.56 for immune reconstitution and OR=1.95; 95% CI=1.85-2.05 for viral suppression), compared to Black PWH. These findings were relatively unchanged after additionally accounting for socio-ecological factors at the county-level, where Hispanic (OR=1.66; 95%CI=1.50-1.83 for immune reconstitution; OR=2.16; 95%CI= 2.04-2.29 for viral suppression) and White PWH (OR=2.28; 95%CI 2.08-2.51 for immune reconstitution; OR=1.88; 95%CI=1.78-1.98 for viral suppression) were significantly more likely to achieve immune reconstitution and undetectable viral loads (Table 2). Recent year of clinical assessment was associated with higher odds of both outcomes.

**Table 2:** Results of the mixed-effect Bayesian inference-based integrated nested Laplace model showing the association between race/ethnicity, year and HIV clinical outcomes before and after socio-ecological factors were adjusted

	**Individual-level variable adjusted**		**Both individual and socio-ecological factors adjusted**	
	**Odds ratio of immune reconstitution**^**a**^	**Odds ratio of viral suppression**	**Odds ratio of immune reconstitution**^b^	**Odds ratio of viral suppression** ^b^
**Race/ethnicity**				
Black	*Reference*	*Reference*	*Reference*	*Reference*
Hispanic	1.70 (1.54-1.87)	2.18 (2.07-2.31)	1.66 (1.50-1.83)	2.16 (2.04-2.29)
White	2.34 (2.14 -2.56)	1.95 (1.85-2.05)	2.28 (2.08-2.51)	1.88 (1.78-1.98)
Other/unknown	0.92 (0.77-1.26)	1.37 (1.20-1.57)	0.98 (0.77-1.25)	1.36 (1.19-1.55)
**Year**				
2012	*Reference*	*Reference*	*Reference*	*Reference*
2013	1.27 (1.19-1.34)	1.14 (1.09-1.20)	1.28 (1.18-1.39)	1.19 (1.12-1.26)
2014	1.78 (1.69-1.88)	1.93 (1.84-1.20)	1.74 (1.59-1.90)	1.97 (1.84-2.11)
2015	2.31 (2.18-2.44)	2.52 (2.40-2.64)	2.33 (2.14-2.54)	2.61 (2.43-2.79)
2016	2.53 (2.40-2.68)	3.09 (2.95-3.24)	2.50 (2.29-2.74)	3.23 (3.00-3.48)
2017	3.35 (3.17-3.55)	3.64 (3.47-3.81)	3.52 (3.19-3.88)	3.93 (3.63-4.25)
2018	4.20 (3.97-4.45)	4.07 (3.88-4.27)	4.51 (4.05-5.03)	4.65 (4.26-5.08)
2019	5.91 (5.59-6.26)	6.39 (6.07-6.72)	6.61 (5.87-7.43)	7.34 (6.67-8.08)

Findings were derived from Bayesian inference-based integrated nested Laplace approximation. Results are presented as odds ratios and 95% credible intervals

^a^Model included race/ethnicity, measurement year, age at diagnosis, years since HIV diagnosis, and transmission risk category

^b^Models adjusted for race/ethnicity, measurement year, age at diagnosis, years since HIV diagnosis, and transmission risk category, in addition to the following county-level socio-ecological factors: social economic factors rank, county averaged years of potential life lost​, county averaged mentally unhealthy days, low birthweight percentage, percentage of adults reporting binge or heavy drinking, uninsured percentage, ratio of population to primary care physicians, high school graduation rate, and crime rate

### Spatial and temporal findings

The results of the temporal analysis using generalized estimating equation models are shown in Table 3. The ORs represent the racial/ethnic disparity in achieving immune reconstitution and viral suppression for each of the year between 2012 and 2019. Disparities were observed for immune reconstitution and viral suppression for which Black PWH were significantly less likely to achieve these two favorable clinical outcomes compared to White and Hispanic PWH in all years.

**Table 3:** Associations between race/ethnicity and the clinical outcomes of immune reconstitution (top) and viral suppression (bottom) stratified by year in Florida, 2012-2019. Findings were derived from repeated measures generalized estimating equation models

	**Immune reconstitution**							
	**2012**	**2013**	**2014**	**2015**	**2016**	**2017**	**2018**	**2019**
**Race**								
Black	Reference	Reference	Reference	Reference	Reference	Reference	Reference	Reference
Hispanic	1.17 (1.08-1.26)	1.25 (1.16-1.34)	1.26 (1.17-1.34)	1.24 (1.16-1.32)	1.23 (1.15-1.30)	1.20 (1.13-1.28)	1.17 (1.11-1.25)	1.22 (1.15-1.30)
White	1.32 (1.23-1.42)	1.40 (1.31-1.50)	1.42 (1.34-1.51)	1.40 (1.31-1.47)	1.32 (1.25-1.40)	1.37 (1.29-1.45)	1.40 (1.32-1.48)	1.36 (1.28-1.44)
Other/unknown	0.89 (0.73-1.09)	1.03 (0.85-1.24)	0.89 (0.76-1.05)	1.01 (0.87-1.19)	0.92 (0.79-1.07)	1.00 (0.86-1.17)	1.04 (0.89-1.21)	1.04 (0.88-1.22)
	**Viral suppression**							
	**2012**	**2013**	**2014**	**2015**	**2016**	**2017**	**2018**	**2019**
**Race**								
Black	Reference	Reference	Reference	Reference	Reference	Reference	Reference	Reference
Hispanic	1.69 (1.55-1.84)	1.68 (1.55-1.82)	1.66 (1.53-1.79)	1.70 (1.58-1.84)	1.73 (1.60-1.87)	1.93 (1.78-2.09)	1.79 (1.65-1.93)	1.94 (1.78-2.13)
White	1.48 (1.38-1.60)	1.50 (1.40-1.62)	1.62 (1.51-1.74)	1.75 (1.63-1.88)	1.63 (1.52-1.75)	1.64 (1.52-1.76)	1.60 (1.49-1.72)	1.76 (1.62-1.92)
Other/unknown	1.18 (0.96-1.46)	1.17 (0.97-1.44)	1.12 (0.94-1.35)	1.43 (1.19-1.72)	1.24 (1.04-1.49)	1.45 (1.20-1.75)	1.35 (1.12-1.63)	1.35 (1.09-1.67)

Results are presented as odds ratios and 95% confidence intervals. All models adjusted for age at HIV diagnosis, year since HIV diagnosis, and transmission risk

**Fig. 2:**
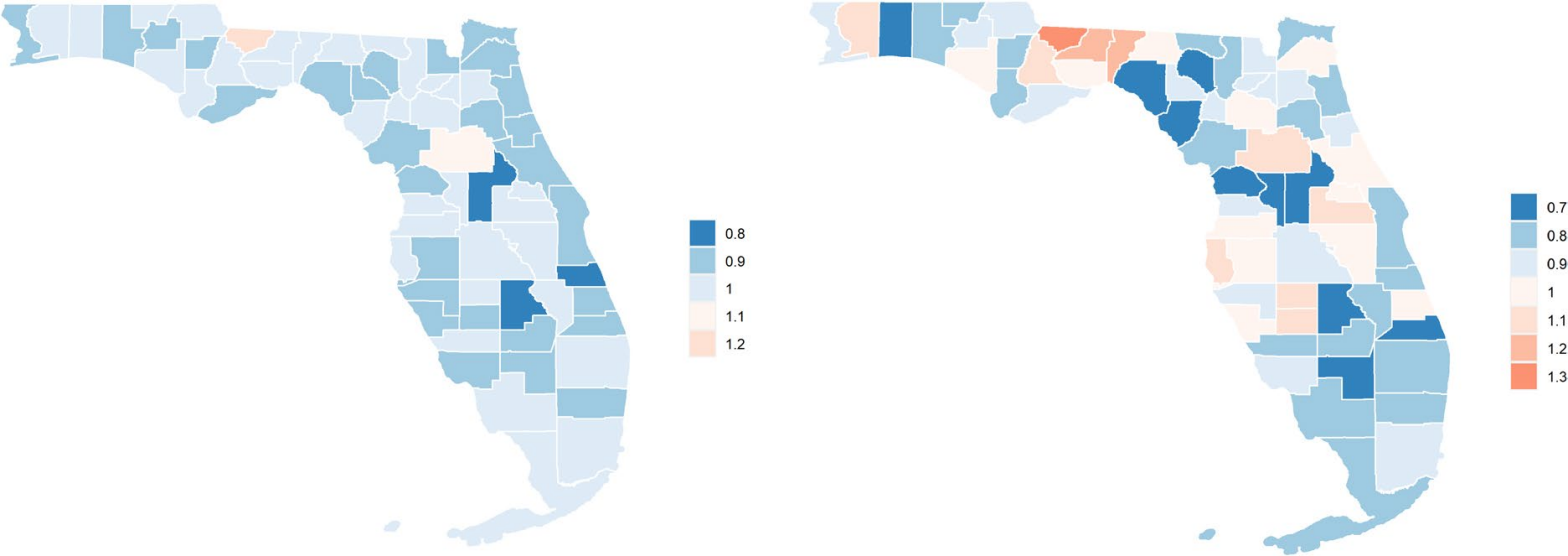
Map of the spatial variations in the relative risk of non-reconstitution (left) and non-suppression (right) among Black vs Non-Black PWH in Florida, 2012 – 2019. Relative risks above 1.0 indicate counties where the risk of non-reconstitution or non-suppression is significantly higher among Black PWH compared to non-Black PWH

The spatial analysis showed that counties where Black PWH consistently fared worse HIV outcomes than White or Hispanic PWH were often located in north-central Florida, including both rural and urban regions (Fig. 2 and supplement Table 1). In some counties located in South Florida (such as Broward and Palm Beach counties), a reverse racial/ethnic disparity can be observed, where Black individuals were more likely than White or Hispanic individuals to achieve immune reconstitution and viral suppression. Detailed relative risk values indicating racial/ethnic disparities in achieving immune reconstitution and viral suppression are included in the supplementary document.

## Discussion

Despite annual improvements in the proportion of Ryan White clients who achieve viral suppression and immune reconstitution, significant racial and ethnic disparities in reaching these milestones remained and with variations by county. These data can be used to better target populations and areas where additional services and support are needed to help achieve the goals set out in the EHE in an equitable manner.

The continued increases in rates of viral suppression found in this study are reflective of trends in the national population of PWH. Steady improvement in viral suppression rates has been reported in the national sample of PWH enrolled in the Ryan White Program, both overall and by race/ethnicity [24]. However, similar to our findings, non-Hispanic White PWH consistently have higher proportions of viral suppression compared to non-Hispanic Black PWH and Hispanic PWH. Samples including PWH who are not enrolled in Ryan White have yielded similar trends in viral suppression and disparities by race/ethnicity in the years after the introduction of integrase inhibitors, although those enrolled in Ryan White tend to have better outcomes than non-enrollees [25]. Individual-level beliefs, behaviors, and conditions can account for a portion of these disparities [26–28]. For example, medical mistrust is more common among non-Hispanic Black PWH and non-Hispanic White PWH, which contributes to disparities in ART adherence and care engagement, key steps in the care continuum to reaching viral suppression [29–31]. While the improvements in viral suppression are encouraging, more work is needed to achieve the ambitious targets set by the EHE and to reach these goals equitably.

In this study, racial and ethnic disparities in viral suppression and immune reconstitution remained after accounting for county-level socio-ecological factors. These findings are consistent with other studies in Florida that found disparities in viral suppression remained between White PWH and Black PWH after adjusting for neighborhood factors [13, 32]. A prior study reported several neighborhood-level factors were associated with viral suppression in Miami-Dade County among Hispanic PWH, but not White or Black PWH [32]. Interestingly, they found there were disparities within more socio-economically advantaged neighborhoods and neighborhoods with both low and high levels of racial and language homogeneity, a proxy for residential segregation. Residential segregation leads to different levels of health care access between predominately Black and predominately White communities and contributes to the disparities in several health outcomes including sexually transmitted infection rates, COVID-19 mortality, and cancer [33–35]. Studies on the impact of segregation on HIV risk outside of Florida have yielded mixed results [36, 37]. Previous studies in Florida studies used ZIP code-level socio-ecological variables, as opposed to county-level variables. Counties are significantly larger geographically and are likely to have a much greater degree of diversity in circumstances [32]. However, results from studies with more granular data are congruous with our findings, supporting the validity of our results.

County-level analyses of disparities between Black and non-Black PWH were found in six counties for immune reconstitution and nine counties for viral suppression. Of the counties where Black PWH were significantly less likely to achieve viral suppression, four of them were identified as priority geographic regions in the EHE (Duval, Pinellas, Hillsborough, and Orange). There were some county characteristics that could be associated with these disparities, such as county-level income inequality by race/ethnicity or degree of residential segregation, but the data were not available for analysis for these CHR variables during this study period. South Florida counties where Black PWH had better clinical outcomes than White and Hispanic PWH were in locations with much higher proportions of Hispanic individuals. Future studies should investigate the determinants of these disparate outcomes to identify the mutable causes that could be targeted to address these disparities.

This study has several limitations. First, our sample of Ryan White enrollees may differ from the overall population of PWH in Florida, since enrollees have to be low income but be able to go through the eligibility verification process every six months. Although efforts have been made to increase the accessibility of Ryan White support, Hispanic PWH are underrepresented in our dataset (24.8% of all PWH but 19.5% of our sample [38]). Second, there may be significant variation within a county in access to care or other socio-ecological variables that influence HIV clinical outcomes. While our results are consistent with other studies that used data from smaller geographic areas, relying on county-level data may lead to biased results due to the ecological fallacy (i.e., relationships present at the population level may not apply at the individual one). For instance, assigning county-level socio-economic status and income indicators to individuals is biased because the within-county distributions of these factors can be highly variable. Individual-level measures of these indicators may better explain some of the disparities identified. Additionally, a limited number of county-level variables were eligible for inclusion in these analyses and some potentially important measures were not included. For example, we were unable to include a direct measure of residential segregation as a variable since this was not added to the CHR until 2016 and was therefore ineligible for inclusion in these analyses. Moreover, our analyses only included the calendar years when the individual had at least one HIV laboratory test. People with long gaps in care engagement may be underrepresented in our analyses. Further, a directed acyclic graph was not constructed to delineate relationships between variables and all included variables were treated as confounders, not mediating or moderating variables, and without a hierarchy to these potential confounders. In the geographic analyses, we classified PWH as Black or non-Black, so we were not able to identify the presence of county-level disparities between other groups (e.g., between Hispanic and White PWH), which may explain the protective association of Black race on the relative risks of non-immune reconstitution and non-viral suppression for some counties. Separate analysis of Hispanic PWH would provide helpful information for developing programs to address disparities in these counties.

## Conclusions

The clinical outcomes of PWH in the Ryan White cohort have improved over time in Florida; however, our findings revealed persistent racial/ethnic disparities in both immune reconstitution and viral suppression over the last decade. While the racial/ethnic gap in immune reconstitution appears stable through time, for viral suppression, it may be growing—particularly for Black PWH. Racial/ethnic disparities in achieving viral suppression were especially prominent for six Florida counties, four of which are priority EHE regions. Our findings revealed persistent, spatially heterogeneous, racial/ethnic disparity in the odds of immune reconstitution and viral suppression in Florida. Only a small portion of the effect of race/ethnicity on clinical outcomes could be explained by available socio-ecological factors, suggesting systemic disparity, as well as warranting the search for additional, modifiable factors from individual and societal spheres to improve HIV outcomes among Black and Hispanic PWH. Further analysis of the underlying factors likely driving these findings, including health care inequities, stigma, and distrust in medicine and public health, along with improved data linkage to achieve better granularity and integration with individual-level factors, will be important for ultimately achieving HIV elimination goals in Florida.

### Supplementary Information

**Supplementary file 1: Supplemental Table 1.** Variations in the relative risk of non-suppression among Black vs Non-Black PWH by Florida county, 2012-2019.

## Data Availability

The data is not publicly available. Reasonable data access request can be made by submitting an application to the FDOH Bureau of Communicable Diseases. More information can be found at https://www.floridahealth.gov/diseases-and-conditions/disease-reporting-and-management/disease-reporting-and-surveillance/data-and-publications/data-requests.html and/or via email DCHPDataRequest@FLHealth.gov.
