# Supplementary material for: Spatial and temporal analysis of HIV clinical outcomes in Florida reveals counties with persistent racial and ethnic disparities during 2012-2019
Source: BMC Public Health. 2024 Mar 9;24:749. doi: 10.1186/s12889-024-17944-w (PMC10924316; doi:10.1186/s12889-024-17944-w)
Supplement: Supplementary file 1 — Supplemental Table 1. Variations in the relative risk of non-suppression among Black vs Non-Black PWH by Florida county, 2012-2019. [file 12889_2024_17944_MOESM1_ESM.docx]

**Supplemental Table 1**. Variations in the relative risk of non-suppression among Black vs Non-Black PWH by Florida county, 2012-2019.

| **County** | **Relative risk of incomplete immune reconstitution** | **Relative risk of non-viral suppression** |
| --- | --- | --- |
| Alachua | 1.08 (1.01-1.16) | 1.09 (1.01-1.19) |
| Baker | 1.05 (0.93-1.18) | 0.95 (0.75-1.20) |
| Bay | 1.07 (0.98-1.18) | 1.04 (0.89-1.22) |
| Bradford | 1.04 (0.92-1.18) | 0.97 (0.79-1.18) |
| Brevard | 0.93 (0.87-0.99) | 0.86 (0.78-0.94) |
| Broward | 0.87 (0.84-0.91) | 0.84 (0.80-0.89) |
| Calhoun | 1.08 (0.96-1.23) | 0.80 (0.61-1.05) |
| Charlotte | 0.98 (0.88-1.10) | 0.85 (0.68-1.05) |
| Citrus | 1.08 (0.94-1.25) | 0.72 (0.55-0.96) |
| Clay | 1.01 (0.93-1.11) | 0.97 (0.78-1.19) |
| Collier | 1.00 (0.92-1.09) | 0.87 (0.77-0.99) |
| Columbia | 1.11 (1.02-1.22) | 0.88 (0.75-1.04) |
| De Soto | 1.00 (0.90-1.11) | 1.18 (0.99-1.42) |
| Dixie | 1.10 (0.94-1.30) | 0.78 (0.59-1.04) |
| Duval | 1.01 (0.97-1.06) | 1.08 (1.03-1.13) |
| Escambia | 0.96 (0.88-1.05) | 0.95 (0.86-1.04) |
| Flagler | 1.00 (0.85-1.16) | 0.98 (0.77-1.26) |
| Franklin | 1.08 (0.90-1.29) | 0.94 (0.69-1.28) |
| Gadsden | 1.15 (1.05-1.26) | 1.32 (1.17-1.49) |
| Gilchrist | 1.10 (0.97-1.25) | 0.92 (0.66-1.27) |
| Glades | 1.01 (0.91-1.11) | 0.86 (0.64-1.14) |
| Gulf | 1.08 (0.93-1.26) | 0.86 (0.68-1.07) |
| Hamilton | 1.12 (0.95-1.32) | 0.82 (0.65-1.03) |
| Hardee | 0.99 (0.88-1.12) | 1.13 (0.90-1.42) |
| Hendry | 1.01 (0.94-1.10) | 0.71 (0.58-0.87) |
| Hernando | 1.10 (0.95-1.27) | 0.97 (0.74-1.27) |
| Highlands | 1.00 (0.90-1.11) | 0.63 (0.51-0.78) |
| Hillsborough | 0.98 (0.94-1.02) | 1.08 (1.03-1.13) |
| Holmes | 1.05 (0.88-1.25) | 0.85 (0.65-1.10) |
| Indian River | 0.99 (0.88-1.12) | 0.81 (0.70-0.95) |
| Jackson | 1.09 (0.99-1.19) | 0.99 (0.86-1.15) |
| Jefferson | 1.09 (0.94-1.26) | 1.21 (0.99-1.48) |
| Lafayette | 1.11 (0.96-1.29) | 0.96 (0.71-1.30) |
| Lake | 1.02 (0.94-1.10) | 0.79 (0.69-0.90) |
| Lee | 0.97 (0.92-1.03) | 0.94 (0.87-1.01) |
| Leon | 1.04 (0.96-1.12) | 1.30 (1.22-1.38) |
| Levy | 1.09 (0.96-1.25) | 0.82 (0.62-1.10) |
| Liberty | 1.08 (0.96-1.22) | 1.13 (0.87-1.48) |
| Madison | 1.13 (1.01-1.26) | 1.03 (0.84-1.27) |
| Manatee | 0.95 (0.87-1.03) | 0.97 (0.85-1.11) |
| Marion | 1.09 (1.01-1.18) | 1.17 (1.07-1.29) |
| Martin | 1.05 (0.96-1.14) | 0.79 (0.65-0.94) |
| Miami-Dade | 1.01 (0.98-1.04) | 0.98 (0.94-1.01) |
| Monroe | 0.85 (0.76-0.94) | 0.89 (0.72-1.08) |
| Nassau | 1.03 (0.85-1.24) | 0.88 (0.67-1.17) |
| Okaloosa | 0.85 (0.75-0.95) | 0.77 (0.63-0.95) |
| Okeechobee | 1.02 (0.93-1.11) | 0.83 (0.66-1.06) |
| Orange | 1.04 (1.00-1.08) | 1.14 (1.10-1.19) |
| Osceola | 1.00 (0.93-1.08) | 1.08 (0.91-1.28) |
| Palm Beach | 1.02 (0.99-1.06) | 0.88 (0.85-0.92) |
| Pasco | 1.01 (0.90-1.12) | 1.01 (0.83-1.24) |
| Pinellas | 1.02 (0.96-1.08) | 1.19 (1.11-1.27) |
| Polk | 1.02 (0.97-1.07) | 0.93 (0.87-1.00) |
| Putnam | 1.02 (0.93-1.11) | 0.87 (0.76-1.01) |
| Santa Rosa | 0.90 (0.75-1.09) | 1.17 (0.91-1.51) |
| Sarasota | 0.89 (0.82-0.97) | 1.03 (0.90-1.17) |
| Seminole | 1.00 (0.91-1.10) | 1.10 (0.98-1.23) |
| St Johns | 1.02 (0.92-1.12) | 0.88 (0.74-1.05) |
| St Lucie | 1.05 (0.98-1.14) | 1.08 (1.01-1.15) |
| Sumter | 1.06 (0.97-1.16) | 0.77 (0.63-0.94) |
| Suwannee | 1.12 (0.98-1.28) | 0.77 (0.60-1.01) |
| Taylor | 1.11 (0.95-1.30) | 0.80 (0.64-1.00) |
| Union | 1.06 (0.96-1.18) | 0.95 (0.84-1.09) |
| Volusia | 0.96 (0.90-1.02) | 1.03 (0.94-1.13) |
| Wakulla | 1.07 (0.92-1.25) | 1.05 (0.82-1.35) |
| Walton | 1.00 (0.86-1.17) | 0.84 (0.63-1.12) |
| Washington | 1.05 (0.90-1.23) | 0.94 (0.76-1.17) |

Results are presented as odds ratios and 95% confidence intervals.

Counties with significantly high odds of achieving immune reconstitution were Columbia (RR=1.11, CI=1.02-1.22), Marion (RR=1.09, CI=1.01-1.18), Gadsden (RR=1.15, CI=1.05-1.26), Alachua (RR=1.08, CI=1.01-1.16), Madison (RR=1.13, CI=1.01-1.26), Orange (RR=1.04, CI=1.00-1.08). These findings did not differ significantly by year. Counties with significantly high odds of a racial disparity in achieving viral suppression were Duval (RR=1.08, CI=1.03-1.13), Pinellas (RR=1.19, CI=1.11-1.27), Hillsborough (RR=1.08, CI=1.03-1.13), Marion (RR=1.17, CI=1.07-1.29), Leon (RR=1.30, CI=1.22-1.38), Gadsden (RR=1.32, CI=1.17-1.49), Alachua (RR=1.09, CI=1.01-1.19), Orange (RR=1.14, CI=1.10-1.19), and St. Lucie (RR=1.08, CI=1.01-1.15).

Counties with lower relative risks of non-immune reconstitution were Brevard (RR=0.93, CI=0.87-0.99), Broward (RR=0.87, CI=0.84-0.91), Monroe (RR=0.85, CI=0.76-0.94), Okaloosa (RR=0.85, CI=0.75-0.95), Sarasota (RR=0.89, CI=0.82-0.97). Counties with lower relative risks of non-viral suppression were Brevard (RR=0.86, CI=0.78-0.94), Broward (RR=0.84, CI=0.80-0.89), Citrus (RR=0.72, CI=0.55-0.96), Collier (RR=0.87, CI=0.77-0.99), Hendry (RR=0.71, CI=0.58-0.87), Highlands (RR=0.63, CI=0.51-0.78), Indian River (RR=0.81, CI=0.70-0.95), Lake (RR=0.79, CI=0.69-0.90), Martin (RR=0.79, CI=0.65-0.94), Okaloosa (RR=0.77, CI=0.63-0.95), Palm Beach (RR=0.88, CI=0.85-0.92), and Sumter (RR=0.77, CI=0.63-0.94).
